# Virulence Attributes and Hyphal Growth of C. neoformans Are Quantitative Traits and the *MATα* Allele Enhances Filamentation

**DOI:** 10.1371/journal.pgen.0020187

**Published:** 2006-11-17

**Authors:** Xiaorong Lin, Johnny C Huang, Thomas G Mitchell, Joseph Heitman

**Affiliations:** Department of Molecular Genetics and Microbiology, Duke University Medical Center, Durham, North Carolina, United States of America; North Carolina State University, United States of America

## Abstract

Cryptococcus neoformans is a fungal human pathogen with a bipolar mating system. It undergoes a dimorphic transition from a unicellular yeast to hyphal filamentous growth during mating and monokaryotic fruiting. The traditional sexual cycle that leads to the production of infectious basidiospores involves cells of both α and **a** mating type. Monokaryotic fruiting is a modified form of sexual reproduction that involves cells of the same mating type, most commonly α, which is the predominant mating type in both the environment and clinical isolates. However, some **a** isolates can also undergo monokaryotic fruiting. To determine whether mating type and other genetic loci contribute to the differences in fruiting observed between α and **a** cells, we applied quantitative trait loci (QTL) mapping to an inbred population of F_2_ progeny. We discovered that variation in hyphal length produced during fruiting is a quantitative trait resulting from the combined effects of multiple genetic loci, including the mating type *(MAT)* locus. Importantly, the α allele of the *MAT* locus enhanced hyphal growth compared with the **a** allele. Other virulence traits, including melanization and growth at 39 °C, also are quantitative traits that share a common QTL with hyphal growth. The Mac1 transcription factor, encoded in this common QTL, regulates copper homeostasis. *MAC1* allelic differences contribute to phenotypic variation, and *mac1*Δ mutants exhibit defects in filamentation, melanin production, and high temperature growth. Further characterization of these QTL regions will reveal additional quantitative trait genes controlling biological processes central to fungal development and pathogenicity.

## Synopsis

Pathogenic fungi have emerged as a major threat to human health. Because most invasive fungi reside in nature and are acquired by inhalation of infectious spores, understanding spore production may uncover strategies to block these infections. Like animals, the fungus Cryptococcus neoformans typically requires two individuals with opposite mating types/sexes, α and **a**, for sexual reproduction and spore generation. However, because the α mating type predominates in nature, C. neoformans has evolved an alternative mechanism, “monokaryotic fruiting,” for individual strains, mostly α, to produce spores. To investigate the genetics of this process, Lin and colleagues analyzed fruiting and two other virulence traits, growth at 39 °C and melanin production in an inbred population. By correlating these phenotypes among the population with the inheritance of DNA markers, the authors showed that (1) strains of either mating type are capable of initiation of fruiting, (2) the α mating type enhances hyphal growth during fruiting, (3) all three phenotypes are affected by gene(s) in the same genomic region, and (4) the Mac1 copper transcription factor encoded by this region was confirmed to affect these properties. This is to the authors' knowledge the first study to dissect complex virulence traits in a human fungal pathogen by mapping genomic loci associated with polygenic traits.

## Introduction

Cryptococcus neoformans is a cosmopolitan fungal pathogen that causes meningoencephalitis in both immunocompromised and immunocompetent hosts [1]. Infection is typically dormant, but can be reactivated when the host immune system is impaired, and the organism then disseminates hematogenously to cause fatal systemic infections with a predilection for the central nervous system [1–4].

*Cryptococcus* infection is acquired by inhalation of airborne propagules from an environmental source [5–7]. Basidiospores have been considered the infectious propagules because they are highly pathogenic, resistant to desiccation and nutrient deprivation, easily aerosolized, and are of an ideal size to penetrate and lodge in the alveoli of the lung [8–12]. C. neoformans normally grows as a budding yeast in nutrient-rich medium or host tissue, but under nutrient limiting conditions, the fungus switches to either dikaryotic hyphal growth (two parental nuclei per hyphal compartment) during traditional sexual reproduction between **a** and α cells [13,14] or monokaryotic hyphal growth (one nucleus per hyphal compartment) during fruiting of cells of the same mating type *(MAT),* most commonly α [8,15,16]. Both pathways lead to hyphal growth and basidiospore production and may thereby affect the distribution of C. neoformans in nature and the pathogenesis of cryptococcosis [17,18]. Because greater than 99% of clinical and environmental isolates are α [1,19] and monokaryotic fruiting is commonly observed among α isolates, the production of basidiospores through monokaryotic fruiting might explain the predominance of α over **a** cells in nature [8,16]. Indeed, a pair of congenic strains (JEC20**a** and JEC21α) that differs only at the *MAT* locus exhibits differences in fruiting: only the α strain (JEC21) is filamentous [8]. In addition, analysis of the progeny from a cross between JEC20**a** and JEC21α showed that only the α progeny produced hyphae [8]. However, monokaryotic fruiting was recently reported to occur in **a** strains independent of the *MAT* locus [20]. Nevertheless, monokaryotic fruiting is less common among **a** isolates, and in several instances it has been difficult to induce **a** strains to undergo fruiting. Thus, it has been unclear whether the mating type contributes to differences in fruiting behavior observed between α and **a** isolates.

Most morphological phenotypes, including the dimorphic conversion from yeast to hyphal growth, are complex traits influenced by both environmental conditions and genetic background. Multiple pathways have been demonstrated to trigger hyphal development in pathogenic dimorphic fungi such as the plant pathogen Ustilago maydis [21–25] and the human pathogen Candida albicans [26–30]. Genetic analysis of these complex traits is challenging due to gene–gene and gene–environment interactions, genetic polymorphism, and variable penetrance. Among organisms with defined sexual cycles, quantitative trait locus (QTL) mapping is a powerful forward genetic approach to track multigenic traits by identifying specific genomic regions that influence these properties [31]. The ultimate goal of QTL analysis is to identify specific gene variants within those regions that affect quantitative variation in the phenotype [32].

The aim of this study was to resolve the conflicts regarding the role of mating type in fruiting behavior [8,20] and to determine whether fruiting is a multigenic trait to which the *MAT* locus contributes. We have shown that hyphal growth during fruiting involves two steps: initiation and maintenance, and both steps are multigenic complex traits. The *MAT* locus is not linked to the initiation of hyphal formation, but it is one of the most significant QTLs governing sustained hyphal growth, establishing a clear link between hyphal growth and the α allele of the *MAT* locus during fruiting. We have also applied QTL mapping to other virulence traits besides filamentation: melanin production and growth at high temperature. We discovered that a common QTL outside of the *MAT* locus governs these three disparate traits. One candidate gene, *MAC1,* was investigated by targeted gene disruption and allelic exchange experiments. These findings provide a genetic and molecular foundation for the subsequent characterization of fungal morphogenesis and pathogenicity.

## Results

### Fruiting by **a** and α Strains Is Indistinguishable

**Figure 1:**
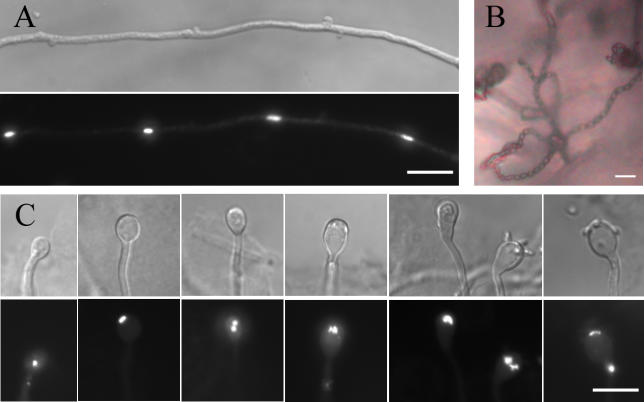
Fruiting in **a** and α Isolates Is Similar The filamentous isolate B3502**a** was cultured on V8 medium in the presence of synthetic α pheromone at 22 °C in the dark for two wk. The filamentous edge far from the original yeast growth patch was separated, fixed, and stained with DAPI. (A) The hyphae produced are monokaryotic with unfused clamps. (B) Four long chains of basidiospores were produced during fruiting. (C) Basidia with one, two, or four nuclei at different stages of basidium development were observed. In (A) and (C), the upper panel shows the DIC images, and the lower panel shows the corresponding fluorescent images. Scale bar, 10 μm.

To determine whether monokaryotic fruiting in **a** isolates is morphologically similar to that observed in α isolates, we examined the filamentous B3502**a** strain that was characterized in a previous study [20]. This strain formed short hyphae and few basidiospores, but in the presence of synthetic α pheromone, hyphal growth and basidiospore production were dramatically enhanced (unpublished data). These observations suggest that both initiation and maintenance of hyphal growth are crucial processes for efficient fruiting. Hyphae and basidia produced at the periphery of the growth patch were fixed and stained with 4′,6-diamidino-2-phenylindole (DAPI) to reveal nuclei. As observed for fruiting of α isolates [8,16,33], the hyphae produced by this **a** isolate showed unfused clamps and contained one nucleus per cell (Figure 1A), both hallmarks of monokaryotic fruiting hyphae. Four chains of basidiospores were produced (Figure 1B), and basidia with one, two, or four nuclei were observed at different stages of basidial development (Figure 1C), similar to the morphology of α isolates. Thus, **a** isolates can be stimulated by pheromone to undergo monokaryotic fruiting that is morphologically similar to α isolates.

### Initiation of Filamentation Is a Complex Genetic Trait Independent of *MAT*

Next, we investigated whether initiation of fruiting is a multigenic inheritable trait and whether or not this process is affected by the *MAT* locus. One previous study proposed that the *MATα* allele was linked to fruiting [8], whereas another report hypothesized that fruiting is controlled by a single locus independent of *MAT*. The latter hypothesis was based on a cross between a filamentous strain and a nonfilamentous strain, from which 20 of 39 progeny were filamentous [20]. However, the limited number of progeny analyzed, combined with an observed greater than 2:1 ratio of α:**a** (14:6) filamentous progeny, is insufficient to exclude an association of mating type with fruiting.

To enable direct comparisons, strain B3502**a**/JEC20**a** that was used in the previous two studies was chosen as one parent for this investigation. A nonfilamentous strain B3501α was chosen as the other parent. The genome sequences for both strains are available. Two hundred twelve F_1_ progeny from the cross between the nonfilamentous strain B3501α and the filamentous isolate B3502**a** were analyzed for mating type and the ability to initiate hyphal formation. The distributions of mating type and fruiting behavior of these progeny are summarized in Table 1. The ratio of the mating types was 1:1 (**a**:α = 102:110), as expected for a single locus exhibiting Mendelian segregation. The progeny population could be classified into four categories based on their ability to initiate hyphal formation: (1) 29% (62/212) of the progeny produced abundant hyphae around the total circumference and were classified as profuse fruiting (the parental filamentous B3502**a** belongs to this group based on this classification); (2) 3% (7/212) of the progeny produced spots of hyphae along the colony periphery and were classified as sparse fruiting; (3) 8% (18/212) of the progeny were inconsistent, producing a few hyphae in some but not all assays and were described as sporadic fruiting; and (4) 59% (125/212) of the progeny produced no hyphae and were classified as nonfruiting. The distribution of each mating type was comparable in all four categories (Table 1), demonstrating that the *MAT* locus is not linked to the ability to form hyphae, consistent with a previous observation [20]. However, in contrast to simple Mendelian (single gene or locus) segregation that was proposed in the previous study [20], in this fairly large progeny set derived from a genetically distinct pair of strains, less than one-third of the progeny (29%) produced abundant filaments, providing evidence that initiation of filamentation is a complex multigenic trait.

**Table 1:**
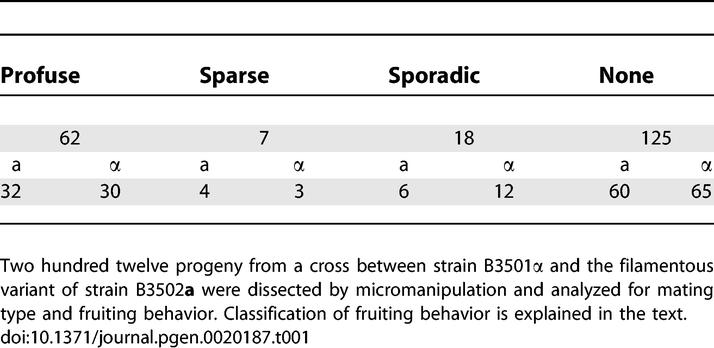
Mating Type and Fruiting Behavior of Progeny between Nonfilamentous Strain B-3501α and the Filamentous Variant of Strain B3502**a**

### Hyphal Elongation Is a Quantitative Trait

**Figure 2:**
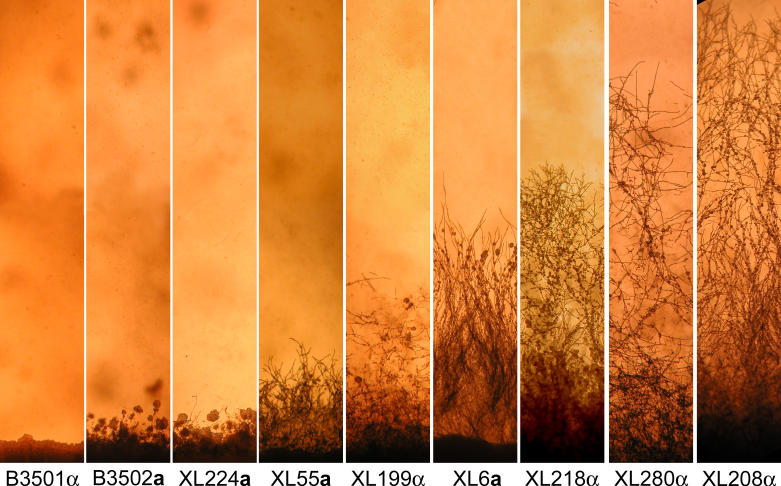
Variation in Filamentous Growth during Fruiting A subset of the fruiting progeny derived from the cross between the nonfilamentous strain B3501α and the filamentous strain B3502**a** was examined microscopically. Cells were cultured on V8 medium for 25 d. Parental strains and several representative progeny displaying variations in hyphal growth are shown with the strain names listed below them.

The developmental steps leading to the production of basidiospores include the requisite initiation of hyphae and maintenance of hyphal growth. From the 212 F_1_ progeny analyzed above, less than a third produced abundant hyphae (Table 1). Surprisingly, not all of them formed the rudimentary short hyphae characteristic of the filamentous parental strain B3502**a** (Figure 2). Instead, a wide range of hyphal phenotypes was observed, and they clearly differed in the rate of hyphal elongation (Figure 2). Here we analyzed QTLs controlling this variation in hyphal elongation.

**Figure 3:**
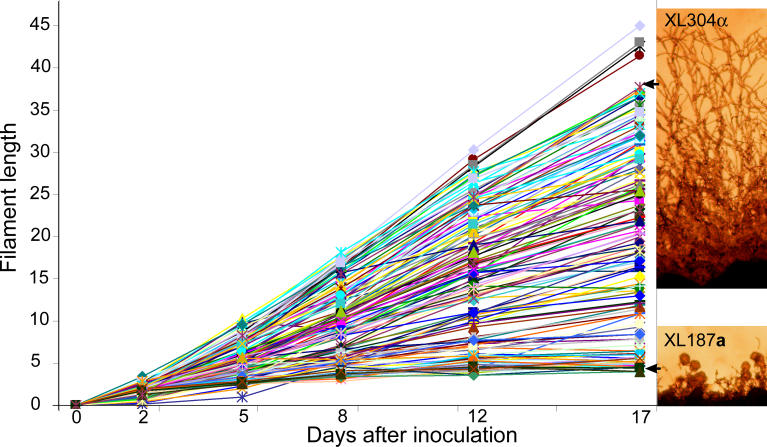
Hyphal Elongation Is a Quantitative Trait (Right panels) Parental strains XL304α and XL187**a** show phenotypic extremes in hyphal growth. Cells were cultured on V8 medium for 15 d. (Left panel) Hyphal length of the progeny shows a continuous trend varying from those that produce hyphae as short as the parental strain XL187**a** or hyphae as long as the parental strain XL304α. Cells were grown on V8 medium and hyphal length was measured at the indicated time from images captured by photo-microscopy.

Because only 29% of the F_1_ progeny were filamentous, to map the QTLs responsible for the variation in hyphal elongation, only a fraction of the population would be informative. Therefore, we chose not to analyze the F_1_ progeny from the cross between B3501α and B3502**a**. Instead, two F_1_ filamentous progeny strains (XL304α and XL187**a**) from the cross between B3501α and B3502**a** that displayed phenotypic extremes were chosen to generate an F_2_ progeny population (Figure 3). Because the F_2_ population was genetically more similar than the F_1_ population, this enabled QTLs to be identified with fewer markers. Strain XL304α produces long hyphae and strain XL187**a** forms only short hyphae (Figure 3), similar to the parental filamentous variant B3502**a** (Figure 2). We dissected 138 meiotic basidiospores from this cross, and all of the F_2_ progeny produced hyphae. This result supports our conclusion that the initiation of filamentation is a genetically inheritable trait; and in this case, all progeny were filamentous when derived from two filamentous parental strains. Similar to the profusely fruiting isolates among the F_1_ progeny, the F_2_ progeny population displayed varied morphological phenotypes, especially with respect to hyphal elongation.

To determine whether hyphal elongation is a quantitative trait, hyphal growth of each progeny was measured periodically. At each time point, digital photomicrographic images of each progeny strain were captured, and hyphal lengths were subsequently determined from these images. Five independent experiments yielded similar results. Hyphal length or growth rate is a consistent, reproducible, and quantitative phenotype, which varied dramatically among the progeny population (Figure 3): some segregants produced short hyphae that did not progress, while other segregants formed long hyphae that extended continuously, and still others were intermediate. The continuous phenotype of filamentous length implies polygenic inheritance amenable to genetic mapping.

### Five Significant QTLs Contribute to the Genetic Variation in Hyphal Growth

**Figure 4:**
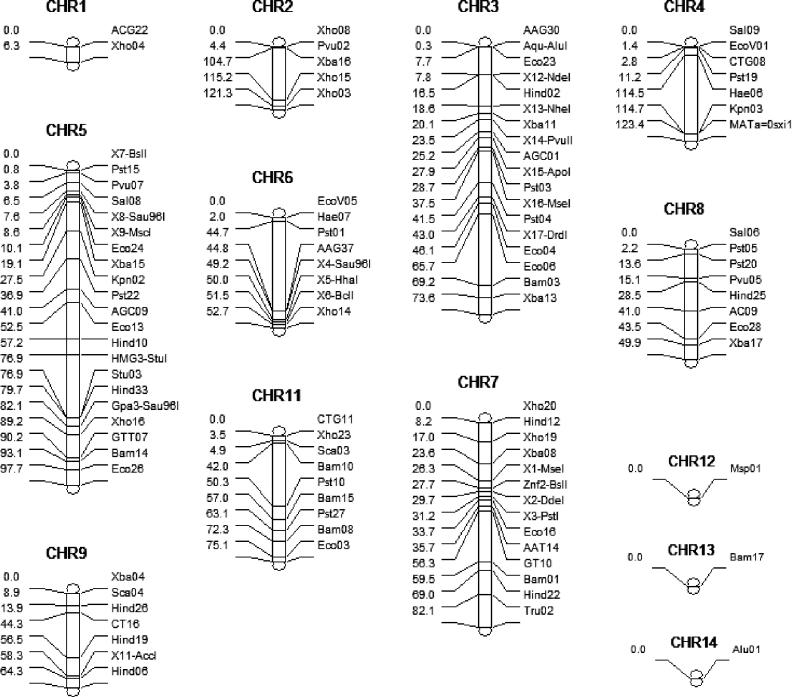
Marker Distribution on Each Chromosome The distance between markers is proportional to the scale and is based on genetic distance calculated from physical distance (see Materials and Methods). The chromosome number is indicated at the top, the marker name is indicated on the right side for each chromosome, and the relative genetic position of each marker is indicated on the left side. The map was generated by WinQTL.

To map the genetic loci mediating variation in this quantitative trait involving hyphal elongation, we randomly selected 47 of the F_2_ isolates of each mating type (94 progeny total) and genotyped them, as well as the parental strains XL304α and XL187**a**. Development of a marker map for this population was necessary to identify QTLs contributing to the phenotypic variation by linkage analysis. The genomes of the closely related progenitor strains B3501α and B3502**a** (congenic with JEC21) are 50% identical [34], and the genomes of the F_1_ parental strains XL304α and XL187**a** are approximately 75% identical based on marker analysis (unpublished data). In addition to existing markers [35], we designed new polymorphic markers to genotype the mapping population. As shown in Figure 4, there were few markers on Chromosomes 1, 12, 13, and 14, and thus these may be uninformative unless QTLs are located close to the few available markers. In addition, Chromosome 10 was not subject to analysis because it was almost entirely monomorphic between the progenitor strains B3501α and B3502**a** [34,35]. Information about the number of markers and their chromosomal locations, the genotypic data, and the phenotypic value (i.e., average hyphal length at 17 d) are available in Table S3. These data were entered into the WinQTL program to perform a genome-wide linkage analysis.

**Figure 5:**
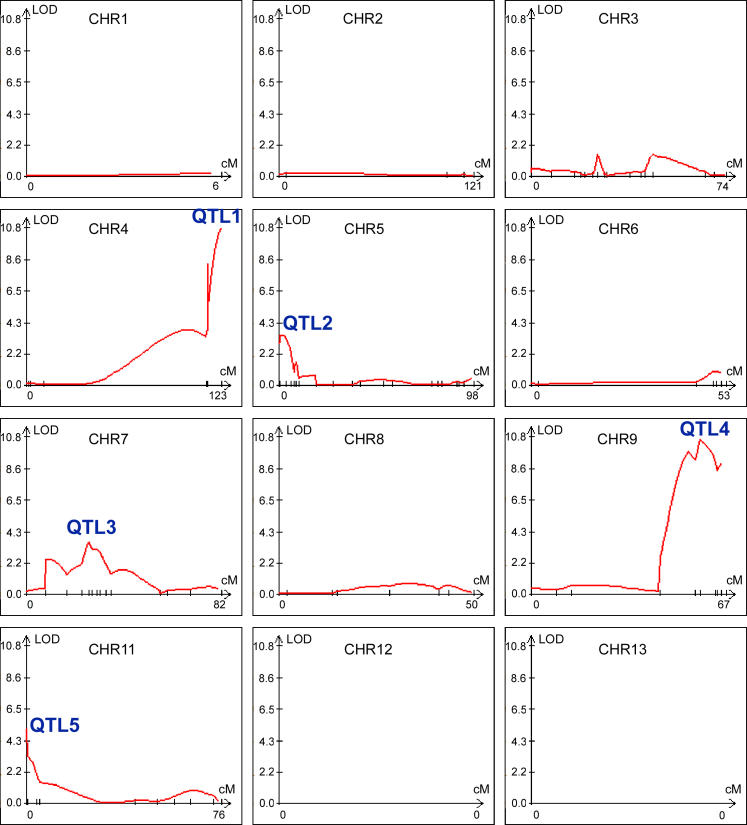
Five Significant QTLs Influence the Variation of Hyphal Elongation Analysis by WinQTL using the CIM method is shown. The *y-*axis shows the LOD score and the *x*-axis shows the marker location on each chromosome. Five QTLs with a LOD score greater than the threshold 3.2 are labeled. Chromosome 14 is not depicted, as no QTL was identified on this chromosome.

To obtain the most robust prediction of QTLs, several mapping methods were applied to search for QTLs, including single marker analysis [36], interval mapping (IM) [37], composite interval mapping (CIM) [38,39], and multiple interval mapping (MIM) [40]. Single marker analysis is based on the association between a marker genotype and trait value. IM uses two observable flanking markers to construct an interval within which to search for QTLs. CIM fits background markers to account for variance caused by nontarget QTLs. MIM uses multiple marker intervals simultaneously to fit multiple putative QTLs. Because each method yielded a similar prediction of the number and location of QTLs, only the results obtained using CIM are shown (Figure 5). In Figure 5, the *y*-axis shows the log of the odds index (LOD) of a particular QTL, indicating the strength of statistical support. The *x*-axis shows the marker distribution along each chromosome. As depicted in Figure 5, there are 5 QTLs with a LOD score above 3.2, the threshold generated through permutation tests, indicating that these QTLs contribute significantly to the genetic variation in hyphal growth observed in this inbred population. Based on the estimation from the CIM analysis, these five QTLs account for 17.1%, 4.4%, 4.6%, 18.8%, and 6.9% of the genetic variance with a cumulative effect of 52%. QTL1 and QTL4 are the two most significant loci accounting for the variation in hyphal growth in this inbred population (17.1% and 18.8%, respectively) and each of the other three QTLs accounts for less than 10% of the effect.

### QTL1 Is the *MAT* Locus and the α Allele Enhances Hyphal Growth during Fruiting

QTL1 is located on Chromosome 4. The region with strong support occurs after position 1,529 kb and extends beyond 1,637 kb. The marker at position 1,529 kb is ~6 kb away from the first gene in the *MAT* locus, and the marker at the peak at 1,637 kb is located within the *SXI1α* / *SXI2**a*** gene, which lies at the opposite end of the *MAT* locus [41]. The region distal to the *MAT* locus has been previously shown to be identical between strains B3501α and JEC21α (congenic with B3502**a**) [34]. Consequently, the *MAT* locus is QTL1 and represents one of the two most significant loci contributing to hyphal growth variation.

To further verify that the *MAT* locus is one of the significant QTLs contributing to the variation in hyphal growth, we separately analyzed the **a** and α isolates of the mapping population. As expected, the *MAT* locus did not appear as a QTL for either subpopulation (unpublished data). The contribution of the *MAT* locus to hyphal elongation is only detectable when the population includes both α and **a** isolates.

**Figure 6:**
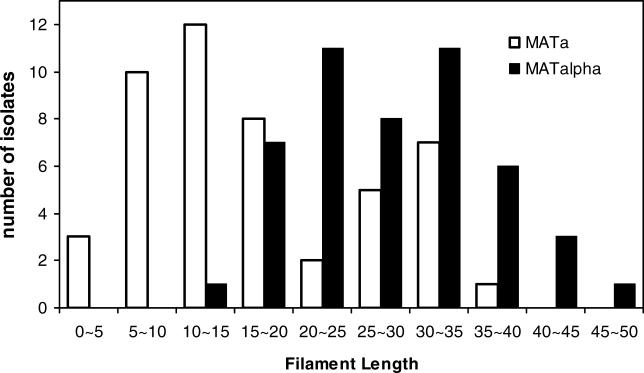
The α Allele of the *MAT* Locus Is Associated with Enhanced Hyphal Growth The number of α or **a** isolates was plotted against the trait value of hyphal elongation to analyze the marker-trait association. Isolates possessing the **a** allele are depicted as open bars and isolates possessing the α allele are depicted as solid bars.

To determine which allele of the *MAT* locus is associated with enhanced hyphal growth, the number of **a** or α isolates was plotted against the trait value of hyphal length to analyze the marker-trait association. As shown in Figure 6, isolates at the two extremes of the phenotype are clearly demarcated. The **a** to α ratio of the progeny producing short hyphae (<15 units) is 25:1, and the **a** to α ratio of progeny producing long hyphae (>35 units) is 1:10. Thus isolates possessing the **a** allele produced shorter hyphae compared with isolates possessing the α allele, and correspondingly the α allele of the *MAT* locus is associated with enhanced hyphal growth.

### Expression Level of Genes in the *MAT* Locus Does Not Correlate with Differences in Hyphal Growth between a and α Isolates

Two hypotheses could explain the correlation of the α allele with better sustained hyphal growth. Either α isolates express to a greater extent genes in the *MAT* locus that promote hyphae growth, or intrinsic differences between the α and **a** alleles are responsible. Unlike most fungi, the *MAT* locus of C. neoformans is unusually large (>100 kb) and encodes more than 20 proteins. Several genes (including the pheromones, pheromone receptors, homeodomain sexual development regulators, and pheromone response pathway elements) in the *MAT* locus are known to regulate dimorphic hyphal development and basidiospore generation [16,20,41–45].

**Figure 7:**
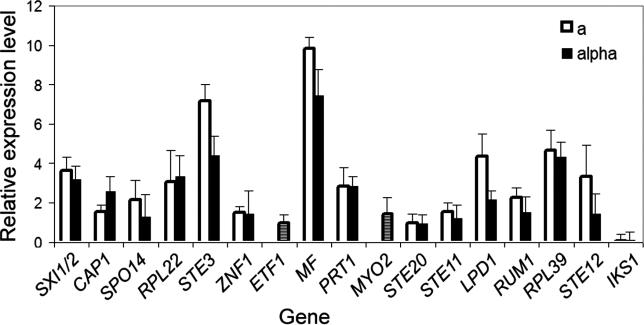
Expression Profile of Genes in the *MAT* Locus Is Similar between **a** and α RNA from strain XL304α and XL187**a** cultured on V8 medium for 24 h was hybridized against each other on a 70-mer genome array after labeling with fluorescent dyes. The expression level was normalized across the genome, and the average of three independent replicates for the genes in the *MAT* locus that are on the array is shown. The **a** alleles are indicated as open bars and the α alleles as solid bars. Because of the high conservation between the **a** and α alleles for the *ETF1* and *MYO2* genes, instead of separate expression levels for each allele, the expression ratio of the two alleles is used and indicated as hatched bars. Because the ratio of *ETF1* and *MYO2* is close to 1, it indicates that the two are expressed at equivalent levels in the two cell types.

To distinguish between the two hypotheses (allele-specific expression or allele-specific function), the expression level of genes in the *MAT* locus at 24 h post-inoculation on V8 medium was examined for the parental strains XL304α and XL187**a**, which display phenotypic difference in hyphal growth. As shown in Figure 7, subtle differences could exist in the expression level of some genes, but the overall expression profile of the **a** or α alleles of the genes in the *MAT* locus was similar. Thus, the steady state expression levels of genes in the *MAT* locus is not likely to be responsible for the phenotypic variation observed for **a** and α isolates in this inbred population, although it remains possible there could be expression differences at the protein level not detected by this approach. Therefore, we favor the hypothesis that differences in the allelic composition likely account for the enhanced hyphal elongation observed with α isolates.

### Association between QTLs of Different Traits: Hyphal Elongation Rate, Growth at Elevated Temperature, and Production of Melanin

Virulence factors in C. neoformans confer survival advantages in both animal hosts and natural reservoirs [46]. For example, C. neoformans hyphae appear to resist killing by environmental predators such as amoebae [47], and the ability to produce hyphae may thus be advantageous in nature. The ability to grow at high temperature (37–39 °C) enables human infection [48], and the production of melanin provides protection from ultraviolet irradiation in the environment and from toxic free radicals generated by host defenses during infection [49]. These virulence properties enable *Cryptococcus* species to be highly successful mammalian pathogens [50–52].

**Figure 8:**
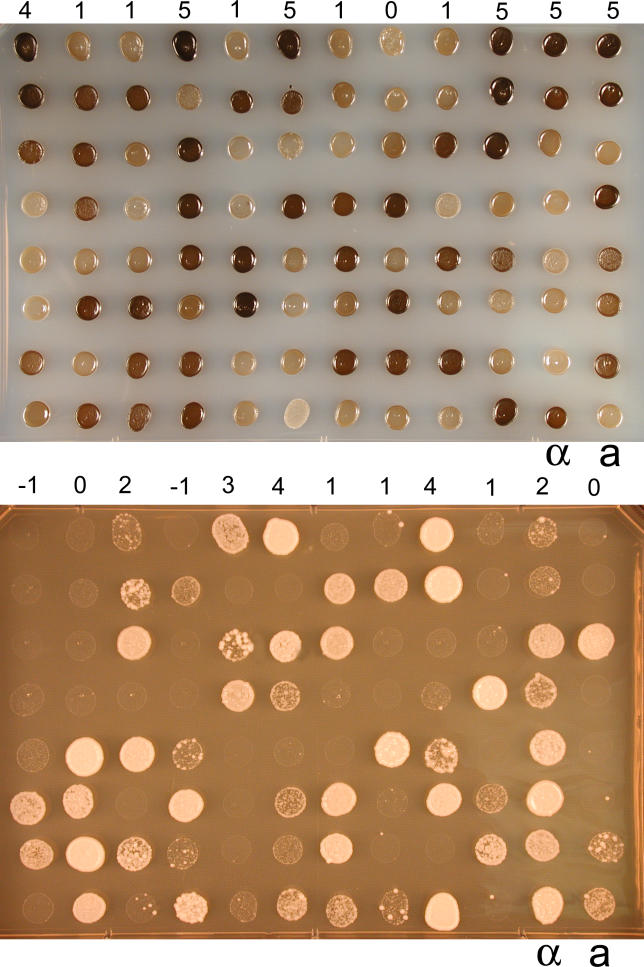
Two Virulence Traits, Melanin Production and High Temperature Growth, Are Quantitative Traits The inbred population generated from strains XL304α and XL187**a** exhibited phenotypic variation in both melanin production (top) and growth at 39 °C (bottom). An equal number of cells were incubated on L-DOPA medium at 22 °C for 4 d (top) and on YPD medium at 39 °C for 4 d (bottom). The numbers above the first row indicate the score assigned to the corresponding progeny in the first row. α and **a** indicate the parental strains XL304α and XL187**a**.

The population generated from strains XL304α and XL187**a** exhibits phenotypic variation in both melanin production and growth at 39 °C, suggesting that these two virulence traits are likely quantitative (Figure 8A and 8B). QTL mapping was applied to identify genomic regions that correlated with these prototypic pathobiological phenotypes.

**Figure 9:**
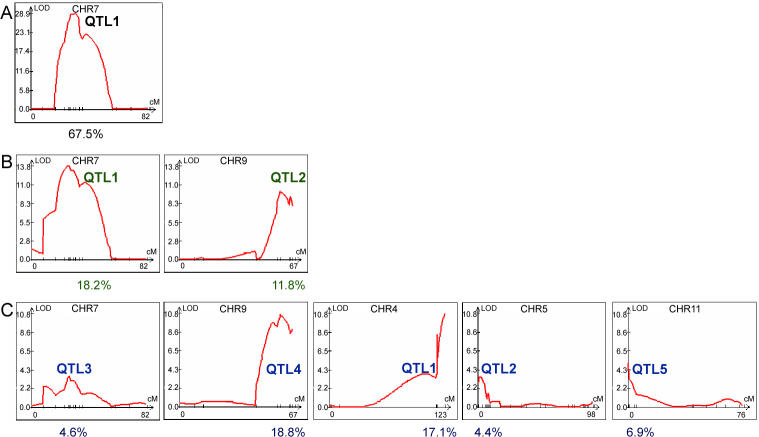
Application of QTL Mapping to Melanin Production and Growth at 39 °C The averaged scores obtained from three independent replicates for melanin production and growth at 39 °C were used as trait values and applied in the WinQTL program. Profiles obtained by CIM are shown. One QTL on Chromosome 7 was identified as responsible for variation in melanin production (A) and two QTLs on Chromosomes 7 and 9 as responsible for growth sensitivity to 39 °C were identified consistently by single marker analysis, IM, CIM, and MIM (B). Other chromosomes without any significant QTL are not shown. Five QTLs responsible for variation of hyphal growth located on Chromosomes 7, 9, 4, 5, and 11 are also shown for comparison (C). The numbers below indicate the percentage of variation accounted for by the corresponding QTLs above according to CIM analyses. LOD threshold for melanin, temperature sensitivity, and filamentation was 7.1, 3.3, and 3.2 respectively.

In *C. neoformans,* oxidation of a variety of diphenolic substrates, including the neurotransmitter L-dihydroxyphenylalanine (L-DOPA), can lead to production of melanin [49]. Variation in the rates of melanization confers variation in the shades of brown pigmentation. We compared the degrees of pigmentation among the mapping population and assigned scores from 0 (no pigment) to 4 (the darkest colonies). Assays of this phenotype were repeated three times, and the scores were averaged and used as trait values. Analysis by the WinQTL program revealed only one QTL on Chromosome 7 (Figure 9A). This single QTL accounted for 67.5% of the total variation observed for this population as estimated by CIM analysis. Given the range of phenotypic variation in a single QTL, it is possible that epigenetic mechanisms affect melanization, that minor QTLs account for some of the variation that was not easily detected, or that undetectable QTLs exist in regions not mapped by our markers.

Similarly, we analyzed growth at 39 °C. A dilution series for each progeny was grown on yeast extract/peptone/dextrose (YPD) medium, and colonies that grew at 39 °C at the lowest cell concentration were given the highest score. Three independent assays were averaged and used as trait values. Two QTLs were consistently identified by all of the methods in the WinQTL program. They were located on Chromosomes 7 and 9 (Figure 9B), and accounted for 18.2% and 11.8%, respectively, of the total observed variation.

There was a surprising overlap among the QTLs for the three phenotypes: hyphal elongation, growth at high temperature, and production of melanin (Figure 9). The QTL on Chromosome 7 is shared by all three traits, and the QTL on Chromosome 9 is shared by high temperature growth and hyphal elongation (Figure 9). Overlapping QTLs for different complex traits have been observed in studies of plants [53–55]. This overlap could be explained by at least two hypotheses that are not mutually exclusive: (1) distinct quantitative trait genes (QTGs) regulate single traits, but they are physically linked—presumably, additional markers in these regions and a larger population size would improve the mapping resolution and dissect these individual QTGs; or (2) the same QTGs have pleiotropic functions and govern multiple pathways.

### *MAC1* on Chromosome 7 Is a Candidate QTG Responsible for Multitrait Variation

**Figure 10:**
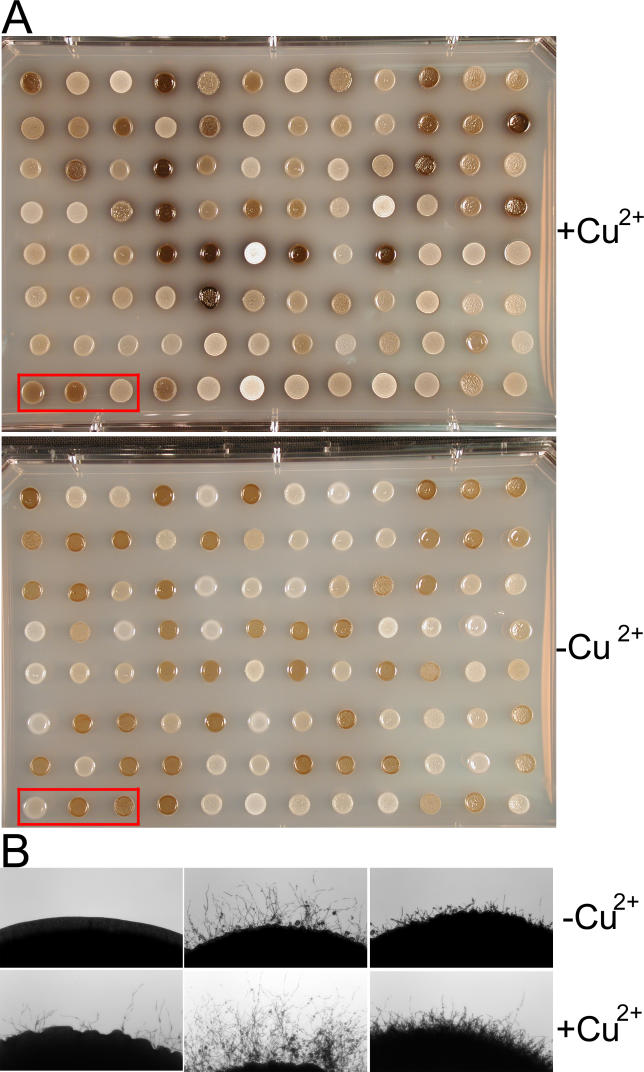
Different Responses to Copper in Melanin Production and Filamentation among the Mapping Population Cells were cultured on L-DOPA medium with or without addition of 50 μM copper sulfate. (A) Differences in melanin production. The upper panel shows the melanin production on medium supplemented with copper and the lower panel without copper addition. (B) Differences in filamentation. The progeny in the left bottom corner of the plate in red rectangles in (A) are shown. The upper panel shows filamentation in the medium without copper and the lower panel with copper addition.

Because the overlapping QTL for three different traits localized to a region on Chromosome 7 (Figure 9), we sought to identify candidate QTGs in this region that could regulate one or more traits. As the region above the threshold LOD on Chromosome 7 is large (~150 kb), we narrowed our search based on two criteria. First, we screened for genes with DNA polymorphisms resulting in amino acid substitutions between the two progenitor strains, B3501α and B3502**a**. These genes are likely QTG candidates, although polymorphisms in regulatory elements might also be responsible. Second, because copper homeostasis is intimately linked to melanization in C. neoformans [56], and the QTL on Chromosome 7 is the main single locus responsible for the variation in melanin production (Figure 9), we tested the response of the mapping population to the addition of copper. The addition of copper to the media caused changes in both melanin production and filamentation to different degrees among the mapping population (Figure 10), suggesting that copper regulation might play pleiotropic roles. Therefore, we searched for genes whose products might control copper responses.

**Figure 11:**
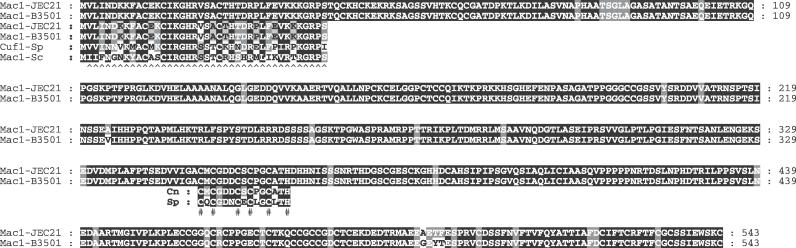
The C. neoformans Mac1 Protein Is Polymorphic Translated amino acid sequences for Mac1 from strains B3501α and JEC21α (congenic to B3502**a**) are depicted. CNG02270 contains a copper-fist DNA binding domain at the N-terminus that is conserved between copper-regulating transcription factors, such as Mac1 of S. cerevisiae and Cuf1 of S. pombe. It also shares a copper-binding motif at the C-terminal region with S. pombe Cuf1 (328–342aa) that is important for activity. ^, sequences underlined with this mark are the predicted copper-Fist DNA binding domain. #, cysteine and histidine residues conserved between Cuf1 of S. pombe and Mac1 of *C. neoformans.* Cn, *C. neoformans;* Sc, *S. cerevisiae;* Sp, S. pombe.

In this region, CNG02270 has been annotated as a putative ligand-regulated transcription factor that contains an N-terminal copper-fist DNA-binding domain (Figure 11). This DNA-binding domain is characteristic of the Mac1 and Ace1 transcription factors in Saccharomyces cerevisiae that regulate cellular copper homeostasis [57]. In addition, the CNG02270 product contains a CXCX_3_CXCX_2_CX_2_H motif at the C-terminal domain (Figure 11). This same motif is critical for responses to copper and modulation of gene expression by the transcription factor Cuf1 of Schizosaccharomyces pombe [58]. We therefore designated CNG02270 as Mac1. Information about CNG02270 can be found at the TIGR Web site under locus name 177.m03070 (http://www.tigr.org/tigr-scripts/euk_manatee/shared/ORF_infopage.cgi?db=cna1&orf=177.t00214).

To determine whether the *MAC1* gene is indeed polymorphic in the mapping population, a restriction fragment length polymorphism (RFLP) marker was designed based on polymorphic sequences between the progenitor strains B3501α and B3502**a**. The RFLP results indicated that the parental strains XL304α and XL187**a** of the mapping population inherited different *MAC1* alleles from B3501α and B3502**a**, respectively (unpublished data). Among the mapping population, isolates with the B3501α allele of *MAC1* exhibited more robust filamentation and growth at 39 °C, but much reduced melanization (Figure S1), indicating that different *MAC1* alleles offer selective advantages under different conditions and that there is a tradeoff between virulence factors. Blast searches of the C. neoformans genome using the full-length Mac1 and Ace1 proteins or their N-terminal copper-fist DNA-binding domains did not retrieve any other homologs, suggesting that there might be only one copper-regulated transcription factor in *Cryptococcus*. This is in contrast to the two transcription factor system in S. cerevisiae where Mac1 and Ace1 independently regulate copper homeostasis under copper-depleted and copper-replete conditions [57].

**Figure 12:**
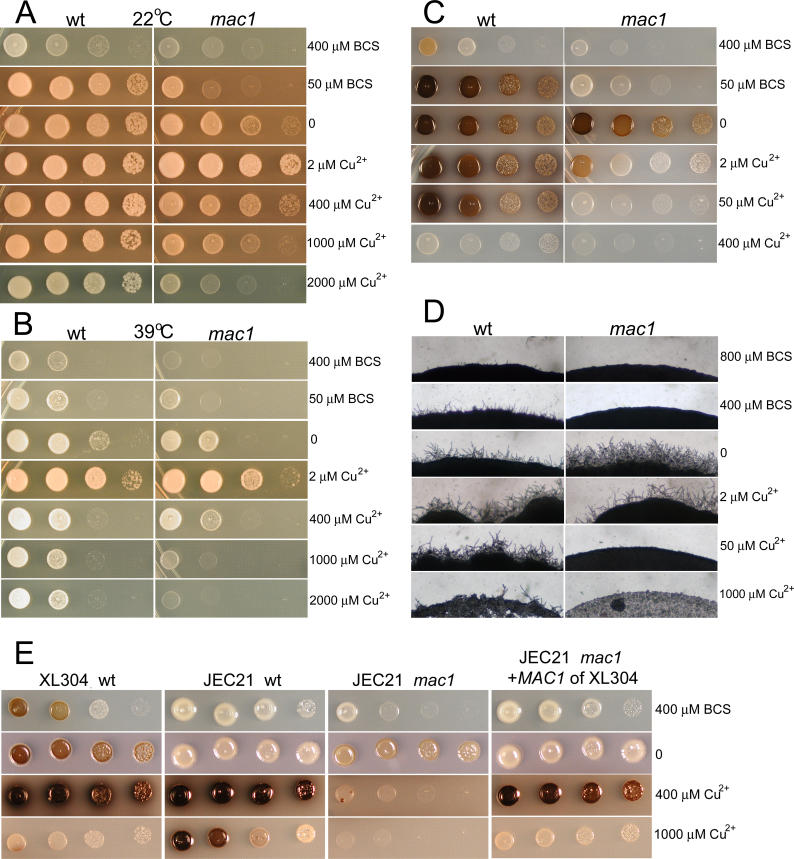
Mac1 Is a QTG Regulating Growth, Melanization, and Filamentation of C. neoformans Wild-type strain XL304α and its *mac1* mutant were cultured on YPD medium with the indicated concentrations of BCS or copper sulfate at 22 °C (A) and 39 °C (B). The *mac1* mutant showed hypersensitivity under copper-limiting and copper-rich conditions at both temperatures. Wild-type and the *mac1* mutant were cultured on L-DOPA medium with the indicated concentrations of BCS or copper sulfate at 22 °C for induction of melanin production (C). The melanin production in *mac1* mutant is also hypersensitive to copper in the medium, showing a bell-shaped response similar to growth (C). Wild-type and the *mac1* mutant were cultured on V8 medium (pH 7.0) with the indicated concentrations of BCS or copper sulfate at 22 °C for induction of filamentation (D). Filamentation of the *mac1* mutant is highly sensitive to copper ions in the medium (D). *MAC1* allele exchange indicates different functions of the two alleles (E). Wild-type XL304α, wild-type JEC21α, JEC21α *mac1* mutant, and transgenic JEC21α *mac1* mutant with the *MAC1* XL304α allele were cultured on L-DOPA medium at 22 °C with the indicated concentrations of BCS or copper sulfate (E).

Deletion of the *MAC1* gene in strain XL304α caused growth hypersensitivity of the mutant under both copper-limiting and copper-rich conditions at 22 °C and 39 °C (Figure 12A and 12B). Thus, Mac1 in C. neoformans appears to maintain copper homeostasis under conditions of copper deficiency or repletion. This effect contrasts with that of S. cerevisiae, in which Mac1 is only active at low concentrations of copper [57]. In addition to yeast growth, melanin production and filamentation of the *mac1* mutant were also hypersensitive to both copper-limiting and copper-enriched conditions, exhibiting a bell-shaped response (Figure 12C and 12D).

Disruption of the *MAC1* gene in two different genetic backgrounds, JEC21α, which inherited the B3502**a** allele of *MAC1,* and XL304α, which inherited the B3501α allele of *MAC1,* rendered both strains highly sensitive in both copper-limiting and copper-rich conditions (Figure 12). To confirm that *MAC1* is a QTG, and that the two different alleles of *MAC1* are not identical in function, the *MAC1* XL304α gene allele was transformed into a *mac1* mutant in the JEC21α background at the native *MAC1* locus. The XL304α allele of *MAC1* complemented the defects of the JEC21α *mac1* mutant under copper-limiting and copper-rich conditions in melanization and growth (Figure 12E and unpublished data). It also conferred some of the properties of the wild-type XL304α strain to this transgenic JEC21 strain with the *MAC1* XL304α gene allele. For example, the transgenic JEC21α was more sensitive to high copper concentrations with respect to melanization than the original JEC21α strain; rather, it responded more like the wild-type XL304α strain (Figure 12E). This allele-exchange experiment indicates that the two alleles of *MAC1* confer different phenotypic effects. Our observations also demonstrate that the effects of Mac1 on growth, melanization, and filamentation are influenced by different genetic backgrounds. Based on these analyses, Mac1 is a likely candidate QTG contributing to the observed phenotypic variation.

## Discussion

Because cryptococcosis originates from environmental sources, hyphal development that leads to the production of highly infectious basidiospores may thus contribute to the epidemiology, ecology, and population structure of C. neoformans in nature. The overwhelming preponderance of mating type α over **a** in clinical and environmental isolates suggests that conventional mating between **a** and α cells that leads to mycelium development and production of comparable numbers of **a** and α progeny is likely to be rare [1,19]. Monokaryotic fruiting observed in α isolates, however, offers a plausible explanation for the distorted distribution of mating types in the population [8,16]. The discovery that some **a** isolates can undergo monokaryotic fruiting challenged the previous hypothesis that fruiting is only associated with the α mating type [20,43]. Our investigations critically reexamined the relationship between mating type and monokaryotic fruiting.

First, we confirmed that monokaryotic hyphae and basidia development is morphologically similar in **a** and α isolates. We observed that efficient development of mycelium leading to basidiospore production requires both efficient initiation of hyphae and sustained hyphal growth, analogous to the initiation and maturation of mycelium in Ashbya gossypii [59]. Mendelian genetic analyses with a large progeny set revealed that the initiation of hyphal growth is independent of mating type and is regulated by multiple genetic loci. However, in contrast to initiation of hyphal formation, mating type is one of the most significant contributing factors regulating sustained hyphal elongation based on QTL mapping of an inbred population. Furthermore, the α allele of the *MAT* locus is associated with enhanced hyphal growth. Analyses of the expression profile of genes in the mating type locus did not reveal substantial differences between the two mating type alleles. Thus, we propose that intrinsic allelic differences between **a** and α mating type genes more likely account for the observed variation in hyphal growth. Thus, by analyzing the fruiting process in two steps, we resolved the conflicts of two previous reports: fruiting is independent of the *MAT* locus at the initiation stage, but efficient and sustainable hyphal growth depends on multiple loci, including the *MAT* locus.

Due to the complexity of the *Cryptococcus MAT* locus, and the diverse pleiotropic functions of its encoded products, the contribution of individual genes to the overall effect of the *MAT* locus on hyphal growth remains to be established. Because the **a** and α alleles of the *MAT* locus are highly divergent and have undergone multiple rearrangements [41], recombination is suppressed within this region, and crossover events in the *MAT* locus have not been observed in the laboratory, which precludes the isolation and analysis of *MAT* recombinant progeny. Therefore, it is challenging to dissect the significant QTGs through QTL fine mapping. Previous studies have shown that several genes in the *MAT* locus are involved in fruiting [45,60,61]. Here we also documented that synthetic α pheromone can enhance fruiting in **a** isolates to form long hyphae and abundant basidiospores. The interactions between multiple genes in the *MAT* locus may orchestrate quantitative effects on hyphal growth and components in the *MAT* locus may interact differently in **a** and α cells. Furthermore, the **a** or α alleles of genes in the *MAT* locus may interact differently with genomic loci outside of *MAT*.

We also demonstrated that QTL mapping can be applied to screen for subtle allelic effects revealing novel loci that affect not only morphogenesis but also virulence traits such as melanin production and growth at elevated temperature. The observation that filamentation, growth at 39 °C, and melanization share common QTLs implies complex interactions among different traits. This association of three phenotypes with the same QTL could be circumstantial; distinct and individual QTGs may be responsible for each phenotype, but the QTGs may be physically linked in the same chromosomal region. Alternatively, the same QTGs in a common QTL may affect multiple cellular pathways and thus have pleiotropic effects. For example, morphological transitions and growth temperature are well-known to be linked in the dimorphic pathogens, including *Histoplasma capsulatum, Penicillium marneffei, Sporothrix schenckii, Blastomyces dermatitidis, Coccidioides immitis,* and Paracoccidioides brasiliensis [62–66]. Furthermore, melanin production, temperature tolerance, and hyphal growth are linked in C. neoformans based on previous observations and studies on *MAC1* reported here [67–69]. As these two hypotheses are not mutually exclusive, both forms of regulation may coexist. Candidate QTGs underlying these QTLs, in addition to *MAC1,* remain to be identified and validated by gene disruption experiments.

The identification of *MAC1* as a candidate QTG for the overlapping QTL affecting filamentation, growth, and melanization illustrates the power of combining QTL mapping with classical mutagenesis experiments. It also supports the notion that some QTGs might have pleiotropic functions, be involved in multiple cellular pathways, and thus affect different traits. The observations that strains exhibited differential sensitivity to copper, and that the effect of disrupting *MAC1* differed depending on their genetic background, indicate that gene functionality is highly influenced by genetic background. Similar observations have been reported for *oxy* mutants of C. neoformans [70] and QTGs affecting temperature sensitivity in *S. cerevisiae* [32].

The mapping strategy used here had specific limitations. First, the inbred F_2_ population shares ≥75% genome identity. This limits the number of QTLs that could be identified for each trait. Theoretically, four times more potential QTLs could exist. Second, the relatively small population size may limit the power to detect QTLs of lesser effect, to dissect linked QTLs, or to analyze the interactions between QTLs. Moreover, the low coverage on Chromosomes 1, 12, 13, and 14 precluded the identification of any QTLs on these chromosomes, which would also lead to an underestimation of their number. Conversely, the close genetic similarity of the population enabled QTLs to be identified with fewer markers. C. neoformans has a relatively high ratio of physical to genetic distances (about 13 kb/cM on average [35]), compared with S. cerevisiae (about 2.7 kb/cM on average according to SGD (http://db.yeastgenome.org/cgi-bin/PGMAP/pgMap)), and thus requires a larger population for fine mapping. This feature of C. neoformans makes it more challenging to precisely map a given QTL and to identify candidate QTGs, although, as we have shown, complementary approaches are advantageous.

This investigation illustrates the value of using nucleotide polymorphisms to construct a genetic linkage map and exploiting the map to parse and identify the genomic locations of loci contributing to phenotypic variations. Successful applications of this approach have been limited to a few model organisms [31,71–74]. With the ever-increasing number of microbial pathogens being sequenced, it will now be feasible to dissect complex, quantitative traits such as morphology- and pathogenesis-related virulence factors through a combination of QTL mapping and functional verification in these pathogenic microorganisms.

## Materials and Methods

### Strains, mating, and isolation of progeny.

The strain B3501α and the filamentous isolate B3502**a** were mated to generate progeny. Strains XL304α and XL187**a** were F_1_ progeny generated from a cross between B3501α and the filamentous B3502**a** isolate. The mapping population consisted of F_2_ progeny and was generated from a cross between strains XL304α and XL187**a**. Information about these strains is summarized in Table S1. Crosses were conducted on V8 juice agar medium (pH 7.0) [75] at 22 °C in the dark. After one to two weeks, individual basidiospores were dissected by micromanipulation. Because the mating medium stimulates monokaryotic fruiting of filamentous strains, in order to harvest mating progeny and avoid spores produced during monokaryotic fruiting, basidiospores were dissected at the far edge from the mating patches. This is based on the observation that dikaryotic mating hyphae elongate faster and more robustly than monokaryotic fruiting hyphae.

### Determination of mating type.

To determine the mating type of the progeny, each isolate was co-cultured with the reference tester strains, JEC20**a** and JEC21α [76] as described above for mating. The isolate and tester strains alone were cultured on the same plate as controls. The mating reactions were examined after 24 h for mating hyphae formation, which signaled the initiation of sexual reproduction. In some cases, the mating type was determined by PCR with *STE20* gene primers that yield a mating type–specific amplicon [77].

### Hyphal formation during fruiting.

Each progeny was cultured on V8 medium (pH 7.0) at 22 °C to induce monokaryotic fruiting hyphal formation. To exclude the effect of opposite mating type pheromone, α and **a** isolates were always cultured on separate plates. Each plate was checked daily for the formation of hyphae, which emanate from the perimeter of the growth patch. The length of the hyphae was determined by placing the plate on a stereoscope at 4× magnification and taking digital images captured with a camera connected to a stereoscope. From the images, hyphal lengths were measured at each time point for each of the progeny clones (1 unit = 60 μm). Cultures were monitored for a period of four weeks. The experiment was repeated five independent times, and at least three independent measurements were taken at each time point for each progeny.

### Microscopy.

Cells were grown on V8 medium on top of glass slides in the dark at 22 °C for 7 d. Hyphae that formed at the perimeter of the colony were fixed in 3.7% formaldehyde in PBS with 1% Triton. Nuclei were visualized by staining with DAPI as described previously [8].

### Melanin production.

An overnight culture of yeast cells was washed twice with distilled water. Cell concentration was determined by absorption at 600 nm. Equal concentrations of cells were spotted on media containing 100 mg/L L-DOPA [78] and incubated at 22 °C in the dark for 2 to 4 d. Melanization was observed as the colony developed a brown color.

### Molecular markers and genotyping.

Markers previously developed for strains B3501α and B3502**a** [35] were screened for polymorphisms between the parental strains XL304 and XL187**a**. Of the screened markers, 69 RFLP and 13 microsatellite markers were polymorphic between the parents and used in the current study. To fill gaps in the map, we used the genome sequences of B3501α and JEC21α to design and confirm an additional 28 polymorphic RFLP markers. Information on all of the primer sequences is summarized in Table S2. The distribution of markers on each chromosome was calculated based on the physical positions of the markers, which were determined using BLAST searches of the two primer sequences for each marker against the *Cryptococcus* genome at the TIGR database Web site (http://www.tigr.org/tdb/e2k1/cna1/)[34]. The genetic distance (in centimorgans) between these markers was calculated as the physical position divided by 13,000 nucleotides, an average distance that was established previously for 1 cM found in C. neoformans strains B3501α and B3502**a** [35]. We genotyped 94 progeny and the parental strains XL304α and XL187**a**. Genotyping conditions were described previously [35].

### QTL analysis.

Genome scans for QTLs were performed for traits including hyphal length, melanin production, and growth at 39 °C using single marker analysis, IM, CIM, and MIM, as implemented by QTL Cartographer (version 2.5) developed by S. Wang, C. J. Basten, and Z.-B. Zeng in the Department of Statistical Genetics and Bioinformatics of North Carolina State University (http://statgen.ncsu.edu/qtlcart/WQTLCart.htm). All analyses were performed using the default settings in the WinQTL program. For CIM, the settings were: walking speed = 2 cM, CIM model = standard model, control marker number = 5, window size = 10 cM, regression method = forward regression method. Permutation tests were performed to generate a threshold LOD (permutation times = 500, significance level = 0.05).

### Culture conditions and RNA preparation.

Equal numbers of yeast cells of XL304α and XL187**a** were spotted on V8 juice medium and incubated at 22 °C in the dark for 24 h. Cells were scraped from the plates and washed in DEPC water. The cell pellet was frozen immediately at −80 °C, lyophilized overnight, and stored at −20 °C until ready for RNA preparation. RNA was prepared using TRIzol reagent (Invitrogen, http://www.invitrogen.com) according to the manufacturer's instructions. The quality of purified RNA was examined on an agarose gel. The experiment was repeated three independent times.

### Microarray hybridization and data analysis.

Fluorescence-labeled cDNA was generated by incorporating amino-allyl-dUTP during reverse transcription of 10 μg of total RNA. Cy3 or Cy5 dye (Amersham, http://www.amersham.com) was coupled to the aminoallyl group as described previously [79]. XL187**a** samples were labeled with Cy3, and XL304α samples were labeled with Cy5 and competitively hybridized to a C. neoformans whole genome 70 mer oligo array with additional serotype-specific and mating-type–specific 70 mer oligos for genes in the *MAT* locus designed by Brian Griffith in the Heitman lab (information about the array can be found at the following Web site: http://genome.wustl.edu/activity/ma/cneoformans). After hybridization as described previously [79], arrays were scanned with a GenePix 4000B scanner (Axon Instruments, http://www.axon.com) and analyzed by using GenePix Pro version 4.0 and BRB array tools (developed by Richard Simon and Amy Peng Lam at the National Cancer Institute; http://linus.nci.nih.gov/BRB-ArrayTools.html). Data were normalized for each array element by dividing the background-corrected median pixel intensity of each spot by the sum of the median intensities of all spots on the array.

### Transformations and disruption of *MAC1*.

To disrupt the *MAC1* gene, an overlap PCR product with the nourseothricin (NAT) resistance marker amplified from plasmid pAI1 [80] and 5′ and 3′ flanking sequences of the *MAC1* locus from strain JEC21α (990 bp and 1012 bp, respectively) was generated using primers JH16814 (GCCGTCACCATCGTGTCC), JH16815 (CTGGCCGTCGTTTTACCAAAGTAGGACACCAGAGCAG), JH16816 (GTCATAGCTGTTTCCTGGGTCCAAATGTTAGGCCC), JH16817 (TCCAATGGTTTCGAGAGATC), M13 Forward (GTAAAACGACGGCCAG), and M13 Reverse (CAGGAAACAGCTATGAC). The overlap PCR product was directly introduced into strains JEC21α and XL304α by biolistic transformation using a Bio-Rad Model PDS-1000/He Biolistic Particle Delivery System, as previously described [81]. Transformants were selected on YPD medium with NAT supplemented with 50 μM CuSO_4_ based on previous studies in S. cerevisiae that *mac1* mutants are auxotrophic for copper [82]. Homologous replacement mutants were screened by PCR using one primer outside of the *MAC1* locus [JH16818 (CCGCCTTTCTCCCATCC)] and one primer in the NAT open reading frame [JOHE117369 (GCGGTTCCGTCGTACAGTGC)]. Disruption mutants were also confirmed by Southern blot. Several independent mutants exhibited similar phenotypes.

### Polymorphisms in *MAC1*.

PCR products of the *MAC1* sequence were generated using primers JH16814 and JH16820 (CTAACATTTGGACCATTCGAT) and genomic DNA from strains B3501α, B3502**a**, XL187**a**, XL304α, JEC21α, and 94 F_2_ mapping progeny as template. The PCR products were then digested with SpeI and subjected to agarose gel electrophoresis to reveal different RFLP patterns for different *MAC1* alleles.

### *MAC1* allele exchange.

An overlap PCR product with the NEO marker from pJAF1 [83] linked to the wild-type *MAC1* gene from strain XL304α was generated using primers JH16814, JH17207 (CTGGCCGTCGTTTTACGCTCAGAAGGGTTCATGTCTG), M13 Forward, and M13 Reverse. The PCR product was introduced into a JEC21α *mac1* mutant by biolistic transformation. Transformants were selected on YPD medium with G418. Integration at the original *MAC1* locus was screened based on loss of NAT resistance. The resulting transgenic strain was confirmed to harbor the desired *MAC1* polymorphism by RFLP.

### Copper sensitivity test.

To test the copper sensitivity of growth, melanin production, and filamentation, wild-type and *mac1* mutant cells were grown overnight in YPD medium supplemented with 2 μM CuSO_4_ (*mac1* mutants are auxotrophic for copper based on this study). Cells were then collected by centrifugation, washed with water, and starved for copper by growing in YPD medium supplemented with 50 μM bathocuproine sulfonate (BCS), a copper-specific chelator. After 8 h, the cells were washed 3× with water and cultured at the indicated temperatures on media with the indicated concentrations of CuSO_4_ or BCS.

## Supporting Information

Figure S1 Association of *MAC1* Allele with Trait Values Association of *MAC1* alleles with filamentation (A), melanin production (B), and high temperature growth (C). The progeny percentile was plotted against the trait values to analyze the marker-trait association. Isolates possessing the B3501/XL304 allele are depicted as open bars and isolates possessing the B3502/XL187 allele as solid bars.
(115 KB DOC)

Table S1 Strains (34 KB XLS)

Table S2 Primer Sequences (27 KB XLS)

Table S3 Marker Location, Genotypic, and Phenotypic Information (185 KB XLS)

## Abbreviations

BCS: bathocuproine sulfonate
CIM: composite interval mapping
DAPI: 4′,6-diamidino-2-phenylindole
IM: interval mapping
L-DOPA: L-dihydroxyphenylalanine
LOD: log of the odds index
*MAT*: mating type
MIM: multiple interval mapping
NAT: nourseothricin
QTG: quantitative trait genes
QTL: quantitative trait loci
RFLP: restriction fragment length polymorphisms
YPD: yeast extract/peptone/dextrose
